# Targeted therapy for metastatic triple negative breast cancer: The next frontier in precision oncology

**DOI:** 10.18632/oncotarget.22580

**Published:** 2017-11-21

**Authors:** Neelima Vidula, Aditya Bardia

**Affiliations:** Aditya Bardia: Massachusetts General Hospital, Boston, MA, USA

**Keywords:** triple negative breast cancer, targeted therapy, androgen receptor, trophoblast antigen 2, immunotherapy

Triple negative breast cancer (TNBC) remains a challenging subtype of breast cancer to treat. Conventionally TNBC has been defined by the absence of the estrogen, progesterone, and human epidermal receptor 2 (HER2) receptors. The current standard of care for treating advanced TNBC remains chemotherapy, albeit often with limited efficacy and poor survival outcomes.

With ongoing molecular research, several new targets have been identified in TNBC including the androgen receptor, phosphoinositide 3-kinase/protein kinase B (PI3K/AKT), trophoblast antigen 2 (trop2), glycoprotein NMB (gpNMB), programmed cell death 1 (PD 1) and programmed death ligand 1 (PD-L1) receptors. In addition, inhibition of poly(adenosine diphosphate-ribose) polymerase (PARP) is appealing in BRCA mutant patients with TNBC. These advances are driving a shift in the classification of TNBC from a cancer classically defined by the absence of receptors to a disease entity that may be defined by the presence of discrete molecular targets, which have the potential to be targeted clinically.

Several promising targeted therapies for metastatic TNBC are emerging, as outlined in Table 1. Targeting the androgen receptor is an exciting area of research, as TNBC expressing the androgen receptor may behave with a less aggressive phenotype (LAR subtype), with a more indolent disease course. A phase II trial explored enzalutamide in androgen receptor positive advanced TNBC [1]. Of 56 patients who tested positive for the androgen receptor using an in-house androgen receptor genomic profiling assay (PREDICT AR), the CBR at 16 weeks was 39% (95% CI: 27-53%) and median PFS was 16.1 weeks.

**Table 1:** Key clinical trials investigating targeted therapies in triple negative breast cancer (TNBC).

Agent	Target	Phase of Clinical Trial	Patient Population	Results
Enzalutamide	Androgen receptor	II	Androgen receptor positive advanced TNBC	In patients with positive PREDICT AR assay, CBR of 39% at 16 weeks (95% CI: 27-53%), median PFS 16.1 weeks
Ipatasertib with paclitaxel	AKT	II	Advanced TNBC, first line treatment	Median PFS of 6.2 months in ipatasertib/paclitaxel arm vs. 4.9 months in paclitaxel/placebo arm (HR 0.60, 95% CI: 0.37-0.98, *p* = 0.037)
Sacituzumab govitecan (IMMU-132)	Trop2	I/II	Heavily pretreated advanced TNBC	ORR 30%, CBR 46%, median PFS 6.0 months (95% CI: 5.0-7.3 months)
Glembatumumab vedotin	Glycoprotein NMB (gpNMB)	I/II	Advanced breast cancer	Median PFS 9.1 weeks in all patients, PFS 17.9 weeks in TNBC, PFS 18.0 weeks in gpNMB positive cancers
Olaparib	PARP	III	Metastatic germline BRCA mutant pre-treated advanced breast cancer	Median PFS 7.0 months in olaparib arm compared with PFS 4.2 months in chemotherapy arm (HR 0.58, 95% CI: 0.43-0.80)
Pembrolizumab	PD-1/PD-L1	Ib	PD-L1 positive advanced TNBC	ORR 18.5%

Similarly, a subset of breast cancers have activation of the phosphoinositide 3-kinase/protein kinase B (PI3K/AKT) pathway. A recent phase II trial randomized patients with advanced TNBC to first line treatment with paclitaxel in combination with ipatasertib, an AKT inhibitor, or placebo (N =62) demonstrated improvement in median PFS in the ipatasertib/paclitaxel arm (6.2 months) compared with 4.9 months in the paclitaxel/placebo arm (HR 0.60, 95% CI: 0.37-0.98, p=0.037), with slightly increased diarrhea and neutropenia in the ipatasertib arm [2].

The trophoblast antigen 2 (Trop2) is another promising target for TNBC. Trop2 is a glycoprotein which may be expressed by many cancers including TNBC and plays a role in cancer cell growth and invasion. Sacituzumab govitecan (IMMU-132) is a novel anti-trop2 antibody drug conjugate which targets trop2 to selectively deliver the active irinotecan metabolite SN-38. In a multicenter trial of heavily pretreated patients with advanced TNBC who administered sacituzumab, the objective response rate (ORR) was 30%, the CBR was 46%, and the median PFS was 6.0 months (95% CI: 5.0-7.3 months), with a median response duration of 8.9 months (95% CI: 6.1-11.3 months) [3]. The FDA has granted sacituzumab a breakthrough drug designation in metastatic TNBC, and a phase III trial with sacituzumab is underway (clinicaltrials.gov#:NCT02574455). Similarly, a phase I/II study of glembatumumab vedotin, an antibody-drug conjugate combining an anti-gpNMB monoclonal antibody with monomethyl auristatin, was studied in 42 advanced breast cancer patients [4]. A median PFS of 9.1 weeks was seen in the entire study population, but the PFS was higher in TNBC patients (17.9 weeks) and those patients with gpNMB-positive tumors (18.0 weeks).

Another compelling novel therapy for advanced breast cancer is olaparib, an oral poly(adenosine diphosphate-ribose) polymerase (PARP) inhibitor. In the randomized phase III trial (Olympiad) comparing olaparib monotherapy to standard chemotherapy in metastatic germline BRCA mutant pre-treated advanced breast cancer, patients receiving olaparib arm had a significantly higher median PFS compared with the chemotherapy arm (7 months versus 4.2 months), yielding a HR of 0.58 (95% CI: 0.43-0.80, *p* < 0.001) [5].

Finally, as immunotherapy continues to be explored in various cancers, it is also being studied in TNBC. Specifically, immunotherapy targeting the PD-1 and PD-L1 pathway is being explored in TNBC. A phase Ib study of TNBC patients with PD-L1 positive advanced disease treated with pembrolizumab, an anti-PD-1 antibody, demonstrated an overall response rate of 18.5% with a median time to response of 17.9 weeks [6], and similar results were reported with atezolizumab. These findings are intriguing as they suggest a possible benefit from immunotherapy which may be long lasting; this is a fascinating direction for TNBC, in which response to therapies is often short lived. Based on these studies demonstrating an initial efficacy of PD-1/PD-L1 blockade in TNBC, additional trials are ongoing exploring immunotherapy combinations which may improve the response to immunotherapy, by making a tumor more immunogenic and susceptible to immune mediated cell death. The advent of immunotherapy is an exhilarating direction for TNBC, as it may have the promise to provide durable responses to therapy.

In summary, there are several targeted therapies for TNBC on the horizon, which promise to shift the paradigm of treating TNBC away from chemotherapy towards therapies geared towards targeting tumor biology. However, genomic complexity, tumor heterogeneity and clonal evolution are major impediments to the success of precision medicine [7, 8], and further research is needed to develop strategies to overcome resistance, including development of rational combination therapies.
